# Human adipose-derived Mesenchymal stem cells, low-intensity pulsed ultrasound, or their combination for the treatment of knee osteoarthritis: study protocol for a first-in-man randomized controlled trial

**DOI:** 10.1186/s12891-020-3056-4

**Published:** 2020-01-15

**Authors:** Mohammad Nasb, Huang Liangjiang, Chenzi Gong, Chen Hong

**Affiliations:** 1 Department of Rehabilitation Medicine, Tongji Hospital, Tongji Medical College, Huazhong University of Science and Technology, Wuhan, 430030 People’s Republic of China; 2 Department of Physical Therapy, Health science faculty, Albaath University, Homs, Syria

**Keywords:** Stem cells, Osteoarthritis, Pain, Low-intensity ultrasound, Knee, Musculoskeletal, Regenerative medicine

## Abstract

**Background:**

Human adipose-derived Mesenchymal stem cells (HADMSCs) have proven their efficacy in treating osteoarthritis (OA), in earlier preclinical and clinical studies. As the tissue repairers are under the control of mechanical and biochemical signals, improving regeneration outcomes using such signals has of late been the focus of attention. Among mechanical stimuli, low-intensity pulsed ultrasound (LIPUS) has recently shown promise both in vitro and in vivo. This study will investigate the potential of LIPUS in enhancing the regeneration process of an osteoarthritic knee joint.

**Methods:**

This study involves a prospective, randomized, placebo-controlled, and single-blind trial based on the SPIRIT guidelines, and aims to recruit 96 patients initially diagnosed with knee osteoarthritis, following American College of Rheumatology criteria. Patients will be randomized in a 1:1:1 ratio to receive Intraarticular HADMSCs injection with LIPUS, Intraarticular HADMSCs injection with shame LIPUS, or Normal saline with LIPUS. The primary outcome is Western Ontario and McMaster Universities Index of OA (WOMAC) score, while the secondary outcomes will be other knee structural changes, and lower limb muscle strength such as the knee cartilage thickness measured by MRI. Blinded assessments will be performed at baseline (1 month prior to treatment), 1 month, 3 months, and 6 months following the interventions.

**Discussion:**

This trial will be the first clinical study to comprehensively investigate the safety and efficacy of LIPUS on stem cell therapy in OA patients. The results may provide evidence of the effectiveness of LIPUS in improving stem cell therapy and deliver valuable information for the design of subsequent trials.

**Trial registration:**

This study had been prospectively registered with the Chinese Clinical Trials Registry. registration number: ChiCTR1900025907 at September 14, 2019.

## Background

Osteoarthritis (OA) is one of the most significant causes of disabilities among people worldwide. OA affects more than 50 million adults in the United States yearly, resulting in a US$100 billion burden on the economy [1–3]. Current conventional therapies, including physiotherapy agents, pharmacologic interventions, and injections, aim to control pain and improve function; however, they remain unsatisfactory [4, 5]. Likewise, invasive procedures, such as arthroplasty, also have limitations [6, 7].

On the other hand, intra-articular injection of mesenchymal stem cells showed promising results in improving the quality of life and prognosis of OA patients [8–10]. This is because the stem cells are able to migrate and attach to the damaged tissues, actively participate in the regeneration of articular cartilages, and reduce the concentration of prostaglandin in the synovial fluid [11–14].

The environment surrounding the cells can significantly affect tissue repair type and quality. Mechanical stimulation plays a key role in enhancing cell proliferation and differentiation [15, 16]. Several biophysical stimulation agents, including but not limited to electromagnetic fields, electric stimulation, and low-intensity ultrasounds, have exhibited promising effects in promoting stem cell proliferation and differentiation [17–20].

Among the available mechanical stimulation tools, the use of LIPUS in regenerative applications has increased sharply in recent years. LIPUS is presently applied in a variety of biomedical applications, such as physiotherapy, drug and gene delivery, bone fracture healing, and tissue repair [21–23]. Moreover, LIPUS is highly useful, because it is noninvasive, safe, and cost-effective.

Earlier studies presented LIPUS irradiation’s effectiveness in promoting osteogenesis and chondrogenic, inducing the differentiation of MSC, and improving cartilage repair in both in vivo and in vitro studies, which summarize in Table 1 [24–30].

**Table 1:** in vitro and in vivo studies of the LIPUS effect on stem cells

First author /Year	Model	Conclusions	Ref.
Ikeda /2006	In Vitro	LIPUS stimulation converts the differentiation pathway of C2C12 cells into the osteoblast and/or chondroblast lineage.	24
Schumann/2006	In vitro	LIPUS could improve in vitro preparation of optimized tissue engineering implants for cartilage repair.	25
Cook /2008	canine	LIPUS improved interface cartilage repair of autologous osteochondral plugs.	26
S.R.Angle /2011	Rat	Ultrasound have a positive effect on osteogenic differentiation of rat bone marrow stromal cells.	27
Ji Hao Cui/2007	Mouse	Ultrasound could be an effective cue to upregulate chondrogenic differentiation of MSCs .	28
Jiang, T /2012	In vitro	LIPUS may induce the osteogenic differentiation of ADSCs in vitro.	29
Jang /2014	In vitro	LIPUS might be used to promote cartilage healing by inducing the migration of CPCs to injured sites.	30
Yamaguchi /2016	Rat	MSC injection with LIPUS is more effective than either treatment in promoting concurrent cartilage repair .	32

*MSC* Mesenchymal stem cells, *ADSC* Adipose stem cells, *CPC* Chondrogenic progenitor cell

Zhou et al. demonstrated that, in addition to accelerating and augmenting the osteogenic differentiation of stem cells, the combination of LIPUS and MSC implantation could prolong the preservation of MSC in vitro [31]. Likewise, Yamaguchi et al. revealed that the combination of the intra-articular injection of MSC and LIPUS showed better results in improving cartilage repair and subchondral reformation in vivo than single treatment [32].

In the present study design, we developed a randomized controlled trial to evaluate the MSC and LIPUS combination among patients with knee osteoarthritis. We hypothesized that the combination of HADMSCs and LIPUS would promote the effectiveness of MSCs in knee cartilage regeneration more than a single-treatment approach.

## Methods

### Study design

**Fig. 1:**
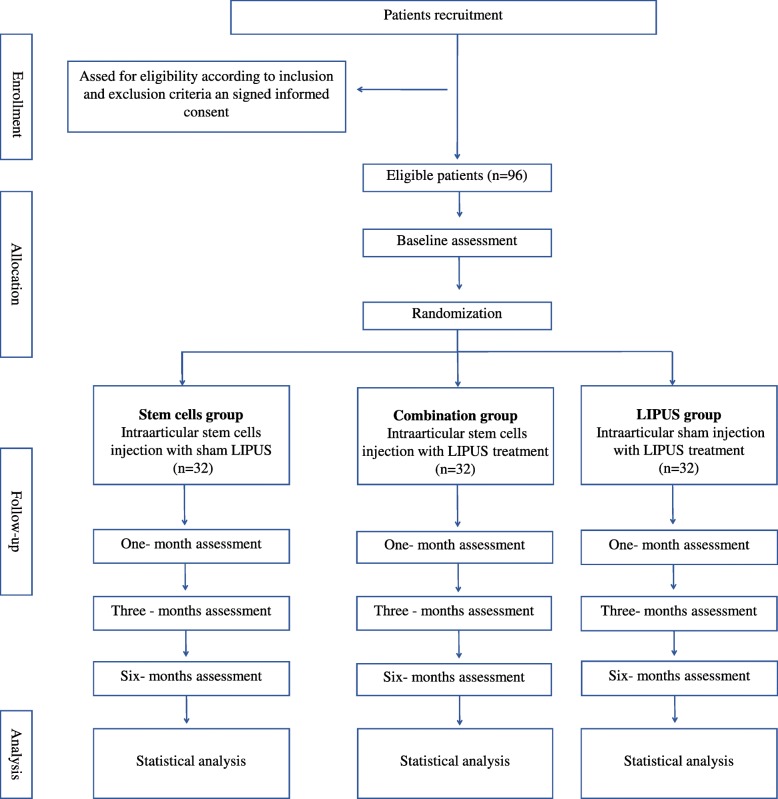
Study flowchart

This is a randomized, placebo-controlled, single-blinded study protocol, designed following the SPIRIT 2013 statement [33]. A summary of the trial is presented in Fig. 1. Patients will be recruited from the Tongji University Hospital (Huazhong University of Science and Technology, Wuhan, China) orthopedic and rehabilitation departments. Study participation will be promoted through the WeChat mobile application, media reports, and advertisements. Participants will undergo preliminary screenings according to the eligibility criteria. Eligible participants will be invited to take the baseline assessment to confirm that they meet the inclusion criteria and provide written informed consent to participate.The current study was approved by the Tongji Hospital ethics committee (TJ-IRB20180901) and prospectively registered with the Chinese Clinical Trials Registry (ChiCTR1900025907). Written informed consent will be obtained before the patients’ enrollment. Evaluators and data collectors will be blinded as to the groups and treatment allocation.

Treatment groups will receive different treatment regimens as follows:

- ❖ Group 1: Single intra-articular injection of 10* 10^6^ HADMSCs with low-intensity pulsed ultrasound treatment for 20 min daily, 5 days a week, for up to 8 weeks.
- ❖ Group 2: Single intra-articular injection of 10* 10^6^ HADMSCs with shame ultrasound irradiation for 20 min daily, 5 days a week, for up to 8 weeks.
- ❖ Group 3: Normal saline with low-intensity pulsed ultrasound treatment for 20 min daily, 5 days a week, for up to 8 weeks.

### Inclusion criteria

- Males and females aged 18 to 70 Years old.
- Radiological diagnosis of osteoarthritis using the American College of Rheumatology criteria.
- Radiological grading of Grade II–III osteoarthritis (OA) of the knee as determined by a qualified radiologist using the Kellgren and Lawrence system.
- Medial or lateral compartment OA .
- Less than 5 degrees varus or valgus knee deformity as measured by the long mechanical axis of the knee on x-ray.
- A minimum pain score of equal or greater than 5/10 on a visual analogue scale (VAS)

### Exclusion criteria

- Previous meniscectomy/significant partial meniscectomy or other knee-related surgery within the last 12 months.
- Previous intra-articular injectable therapies within the last 6 months.
- The patients who have meniscus injury of knee joint need surgical repair.
- The patients who have severe coagulation disorders or cardiopulmonary conditions.
- The women who are pregnant or nursing.
- There are electronic implants such as pacemakers in the body.
- The patients who are infected the HIV, the virus of hepatitis or syphilis or Bleeding disorders.
- The patients with severe cognitive impairment who cannot follow instructions to complete the treatment.
- History of cancer.
- Immunodeficiency patients.
- History of systemic illness or significant organ impairment/failure (i.e., renal failure).
- The patients who have congenital or acquired knee malformation.
- Patients or researchers who are participating in other clinical trials believe that other reasons are not appropriate for clinical trials.

### Randomization

Participants will be randomly allocated to one of the three groups in a ratio of 1:1:1. Patients’ allocations will be according to a randomly generated number using a computerized randomization table (http://www.randomization.com). This table of randomization numbers will be provided only to specified personnel at the stem cell center. Moreover, using identical placebo procedures will ensure blinding and a central automated allocation procedure.

### Blinding

Blinding both participants and healthcare professionals is not feasible in this type of intervention. However, in order to minimize bias, the outcome evaluation will be standardized and done by well-trained investigators. Furthermore, the investigators and evaluators will be blinded to the group/treatment assignment. After completing the study, the statistician will present un-blinded data to the investigators.

### Autologous HADMSC preparation

For this study, the researcher chose human adipose stem cells as the stem cell source. This is because human adipose stem cells can be easily isolated by a minor invasive approach and in larger quantities than bone marrow. Moreover, human adipose stem cells have a chondrogenic differentiation potential [34–36] In this study, all participants will undergo a lateral abdominal approach to abdominal liposuction harvesting. The participants will be given antibiotics as a prophylaxis course 1 day before and 4 days after the liposuction [37].

The procedure includes anesthesia of the lateral abdominal area. The anesthesia solution contains a tumescent fluid that comprises approximately 30–40 ml of (2%) lignocaine and 1 ml of 1/1000 adrenaline. The mixture will be buffered by 1 ml of 8.4% bicarbonate and then suspended in saline solution to a total of 1000 ml.

Up to 60 ml of adipose tissue and tumescent fluid will be exuded using a 4 mm lipo-aspiration canula. The aspirated subject will be placed in a sterile medical grade Shippert TissuTrans Collection filter (Shippert Medical, CO, USA). A certified and well-trained doctor will conduct the liposuction procedure and all participants will be checked after 1 week of cell harvest.

### Mesenchymal stem cells (MSCs) derivation

we will conduct the isolation and expansion procedure by following the method designed by Zuk et al. [38] The aspirated content in the filter will be treated in a fully sterile environment in a Biological Safety Cabinet (Class II) and obeying strict aseptic systems with (ISO 5) air quality at least. Moreover, all the equipment, reagents, and buffer will be sterile, validated, and qualified for cell culture use. Stromal vascular fraction (SVF) will be isolated by enzymatic digestion followed by centrifugation.

Later, cell culturing will be accomplished under hypoxic conditions and using a standard growth medium (10% fetal bovine serum). The cells will be cultured to reach a confluency of about 80% and later passage 2 (P2). Upon reaching P2, the researcher will wash the cells to remove the fetal bovine serum. Then, HADMSCs will be suspended in a clinical-grade MSC cryoprotectant media and cryo-preserved by a clinical-grade cryoprotectant media [39, 40].

The cells will be detected via flow cytometry (FACS), which will be completed independently on all HADMSC samples using the four surface markers for the MSCs, as indicated by the International Society for Cellular Therapy (ISCT) [41]. The researcher will also test the presence of MSC markers (CD44, CD 90, CD105, and CD 73) and the absence of hematopoietic surface markers (CD 34 and CD45). In addition, all HADMSC samples will undergo independent sterility testing for microbial growth or/and contamination.

Dosages including roughly 10* 10^6^ MSCs each will be frozen separately in sterile cryovials in an approved cell cryoprotectant medium using a validated control rate freezing method and kept in liquid nitrogen until needed [40]. On the day of injection, one cryovial will be liquefied at 37 °C using a sterile water bath and then centrifuged to eliminate the cryoprotectant medium and add injectable sterile isotonic (0.9%) normal saline to a total of 3 ml. Cell count and viability will be established through the use of a Muse Cell Analyzer (Merck Millipore, USA).

### Interventions

#### HADMSC intra-articular injection technique

At the time of injection, the patient’s knee will be prepared following the standard sterile procedures. To ensure the accurate intra-articular placement of the needle, the injection will be carried out under continuous ultrasound guidance.

A total of 2 ml of (1%) lidocaine will be injected subcutaneously to reach the knee joint capsule and provide local anesthesia under aseptic settings. Then, roughly 10 million HDAMSCs will be injected into the intra-articular area using the superolateral patella approach. Patients in the sham injection treatment group will be injected an equivalent volume of sterile saline at the same interval of stem cells treatment group.

#### Ultrasound therapy

In the LIPUS treatment patients, LIPUS will be applied using an aqueous gel that does not include any pharmacological substances as a conduction medium. LIPUS will be applied in circular movements with the applicator probe at right angles to achieve maximum absorption of the energy. The treatment areas will include both the tibiofemoral and patellofemoral borders of the target knee on both the medial and lateral margins, making sure to avoid to patella.

Pulsed ultrasonic waves of (1 MHz) frequency and 0.5 w/cm2 intensity will be applied with a 5 cm diameter applicator probe for 20 min (Sonopuls 590, EnrafNonius BV, The Netherlands). On the other hand, patients in the placebo ultrasound treatment will receive the same number of treatment sessions of a similar duration as those in the experimental group. While the sham LIPUS treatment will only use inactive doses of LIPUS with an intensity of 0 W/cm2, along with the gentle application of an inert-gel for 20 min to the treated area [42, 43]. The placebo treatment protocol has been successfully examined in previous studies [44, 45].

Patients recruited to LIPUS will have the treatment once a day, 5 days a week for 8 weeks. All patients will be treated by the same therapist, who was not blinded to the treatment groups as the therapist will set the setting parameters.

#### Outcome assessments

All outcome assessments will be evaluated by well-trained investigators who are blind to the treatment/groups allocation. Moreover, another researcher will perform independent random cross tests. Assessments will be performed at baseline (1 month prior to treatment) and after 1 month, 3 months, and 6 months’ follow-up.

#### Primary outcome measures

The Western Ontario and McMaster Universities Osteoarthritis Index (WOMAC) is a widely-used scale in the assessment of OA. The successful application of WOMAC has proven its validity, reliability, and responsiveness in OA patients. Researchers have conducted a number of empirical studies have been conducted to estimate the MCID and PASS of the WOMAC in patients with OA. A 10-point numeric rating scale of WOMAC will be the primary outcome [46]. The WOMAC score will be normalized to a 100-point scale. In bilateral OA cases, when both knees meet the eligibility criteria, one of them will be treated and the WOMAC index will evaluate the same knee through the study period.

#### Secondary outcome measures

The secondary outcome measures are cartilage thickness measurement, visual analog scale (0 to 100 mm) for pain (VAS), a quality-of-life index (QLI), range of knee motion, muscle strength, global posture analysis system (GPS), and Likert scale. Moreover, the researcher will document safety records, including adverse events and serious adverse events, in addition to interventions and/or co-interventions, such as exercise, yoga, medication, and self-applied massage. Moreover, clinical examinations will be undertaken at the same intervals to document joint line tenderness warmth, effusions, erythema, and limitation in range of motion.

#### Magnetic resonance imaging (MRI)

As Previous studies have shown that tibial-plateau bone area associates with the development of osteoarthritis [47]. A three Tesla-MRI with volumetric 3D gradient and fast spin echo sequences will be performed. Osiris software (Digital Imaging Unit, University Hospital of Geneva, Switzerland) will be used in Image processing. The volume of the cartilage plates (femoral condyles, tibial plateaus and patellar) will be manually isolated from the total volume. Data will be resampled using bi-linear and cubic interpolation for the 3D interpretation, while Osteophytes, will not be involved in the area of interest.

The cartilage plate volume will be calculated by adding values of the pertinent voxels within the resultant binary volume. Moreover, Semi-quantitative measures of cartilage defects will be obtained using a modified International Cartilage Repair Society (ICRS) score as it demonstrated in Table 2.

**Table 2:** modified International Cartilage Repair Society (ICRS) score scale

Modified International Cartilage Repair Society (ICRS) score	
Grade 0	Normal cartilage
Grade 1	Focal blistering and intra-cartilaginous low- signal intensity area with an intact surface and bottom
Grade 2	Irregularities on the surface or bottom and loss of thickness of less than 50%
Grade 3	Deep ulceration with loss of thickness of more than 50%
Grade 4	Full-thickness cartilage wear with exposure of subchondral bone

### Sample size calculation

The current study was designed to achieve 80% power overall to detect a 10-point change on the WOMAC between groups after 6 months (the significance level was set at 0.05). Based on an earlier study using a similar outcome measuring method with adding around 20% for the overall attrition rate. Thus a sample size of 96 (32 per group) will be used for estimating a two-sided 95% confidence interval (CI) [48].

The statistical hypotheses are:

H0: mean WOMAC Index (with LIPUS) = mean WOMAC Index (conventional).

HA: mean WOMAC Index (with LIPUS) ≠ mean WOMAC Index (conventional).

### Statistical analyses

Data will be recorded in the case report form. The outcomes will be presented as the mean ± standard deviation (SD). Data analysis will be done using the SPSS version 24.0 statistical software. Comparing the outcomes between groups will be calculated using analysis of variance (ANOVA) or an alternative nonparametric test if the conditions for parametric tests are not applicable.

## Discussion

Cellular-based treatments for OA are rapidly evolving, in this first-in-man protocol study design, we aim to compare the efficacy of LIPUS and Mesenchymal stem cells combination versus individual approaches. The study will provide data and guidance whether LIPUS-aided regeneration process will show a significant improvement in patients with osteoarthritis. Moreover, it offers an opportunity to develop the trial methodology for the evaluation of the putative role of LIPUS in human regenerative medicine and provide suggestions for the design of the following studies to demonstrate potential efficacy. We will attempt to standardize all treatment and outcomes measurement by performing it at the same time of the day by an instructed and supervised doctor.

To the best of our knowledge, the present study design will be the first randomized clinical trial comparing the efficacy of LIPUS-MSC combination to the individual treatment for the treatment of knee osteoarthritis.

## Data Availability

The data set will be made available upon reasonable request from the corresponding author.

## Abbreviations

GPS: global posture analysis system
HADMSCs: Human adipose-derived Mesenchymal stem cells
ICRS: International Cartilage Repair Society
LIPUS: low-intensity pulsed ultrasound
MSCs: Mesenchymal stem cells
OA: Osteoarthritis
QLI: Quality-of-life index
SVF: Stromal vascular fraction
WOMAC: Western Ontario and McMaster Universities Index of osteoarthritis
